# Global warming potential of farming systems across England: possible mitigation and co-benefits for water quality and biodiversity

**DOI:** 10.1007/s13593-025-01015-4

**Published:** 2025-04-02

**Authors:** Yusheng Zhang, Adrian L. Collins

**Affiliations:** Net Zero and Resilient Farming, Rothamsted Research, North Wyke, Okehampton, Devon EX20 2SB UK

**Keywords:** Greenhouse gas emissions, Agriculture, Best management, Policy, Trade-offs

## Abstract

Agriculture is a key contributor to gaseous emissions causing climate change, the degradation of water quality, and biodiversity loss. The extant climate change crisis is driving a focus on mitigating agricultural gaseous emissions, but wider policy objectives, beyond net zero, mean that evidence on the potential co-benefits or trade-offs associated with on-farm intervention is warranted. For novelty, aggregated data on farm structure and spatial distribution for different farm types were integrated with high-resolution data on the natural environment to generate representative model farms. Accounting for existing mitigation effects, the Catchment Systems Model was then used to quantify global warming potential, emissions to water, and other outcomes for water management catchments across England under both business-as-usual and a maximum technically feasible mitigation potential scenario. Mapped spatial patterns were overlain with the distributions of areas experiencing poor water quality and biodiversity loss to examine potential co-benefits. The median business-as-usual GWP20 and GWP100, excluding embedded emissions, were estimated to be 4606 kg CO_2_ eq. ha^−1^ (inter-quartile range 4240 kg CO_2_ eq. ha−^1^) and 2334 kg CO_2_ eq. ha^−1^ (inter-quartile range 1462 kg CO_2_ eq. ha^−1^), respectively. The ratios of business-as-usual GHG emissions to monetized farm production ranged between 0.58 and 8.89 kg CO_2_ eq. £^−1^ for GWP20, compared with 0.53–3.99 kg CO_2_ eq. £^−1^ for GWP100. The maximum mitigation potentials ranged between 17 and 30% for GWP20 and 19-27% for GWP100 with both corresponding medians estimated to be ~24%. Here, we show for the first time that the co-benefits for water quality associated with reductions in phosphorus and sediment loss were both equivalent to around a 34% reduction, relative to business-as-usual, in specific management catchment reporting units where excess water pollutant loads were identified. Several mitigation measures included in the mitigation scenario were also identified as having the potential to deliver co-benefits for terrestrial biodiversity.

**Supplementary Information:**

The online version contains supplementary material available at 10.1007/s13593-025-01015-4.

## Introduction

Global food production is responsible for ~25% of greenhouse gas (GHG) emissions (Fan et al. 2023). After carbon dioxide (CO_2_), methane (CH_4_) and nitrous oxide (N_2_O) are the second and third most important GHGs globally (Liu et al. 2023). While secondary and tertiary industries dominate global anthropogenic sources of CO_2_, in the case of CH_4_ and N_2_O, agriculture is an important global source (Liu et al. 2023). Atmospheric concentrations of CH_4_ more than doubled between the pre-industrial era and the twenty-first century, while concentrations of N_2_O increased by ~22% (Yang, et al. 2021; Liu et al. 2023). A recent Inter-governmental Panel on Climate Change (IPCC) report has suggested that 44% of methane (CH_4_) and 81% of nitrous oxide (N_2_O) emissions from human activities globally during 2007–2016 could be attributed to agriculture, forestry, and other land use. This represents 23% of the total net anthropogenic emissions of greenhouse gases (GHGs) (IPCC 2019a). These global scale estimates have clearly highlighted the magnitude and distinctive contributions of GHGs from land-based activities. Their effective mitigation will therefore, to a certain degree, determine if we can achieve the ambitious net zero goal to keep the increase in temperatures below 1.5 °C above pre-industrial levels as specified in the Paris Agreement.

Alongside the important contribution of agriculture to global GHG emissions and the climate change crisis, agricultural loads of nutrients to water probably already exceed sustainable limits (Rockström et al. 2009; Boretti and Rosa 2019). Equally, the change in land use associated with agricultural expansion and intensification has driven a massive acceleration in the global loss of biodiversity. Here, up to 30% of all mammal, amphibian, and bird species is threatened with extinction this century (Díaz et al. 2005).

Turning more specifically to the UK, agriculture contributed 10% of GHG emissions in 2018, compared with 7% in 1990, with the increase reflecting slow progress in reducing emissions from key farming sources and accelerated decarbonization in other sectors (Climate Change Committee 2020). In October 2021, the UK Government published its ambitious plan to deliver the legal target for net zero by 2050, with an intermediate target of reducing GHG emissions by 68% relative to 1990 levels, by 2030 (HM Government 2021). In delivering cleaner air, the UK government is also committed to delivering cleaner freshwaters. Rural water quality in the UK has declined relative to pre-1960 levels and diffuse agricultural water pollution remains a significant threat (Whelan et al. 2022). Equally, the latest State of Nature Report for the UK has suggested that the abundance of many terrestrial and freshwater species has declined by 19% since 1970, with a concomitant 13% reduction in the distribution of many invertebrate species (Burns et al. 2023). The specific role of agriculture in the UK in driving biodiversity decline has been highlighted in the work of Burns et al. (2016).

Multiple approaches have been used to link land-based activities with GHG emission quantities and potencies. Controlled experiments are, for example, still being undertaken to examine the role of weather conditions, soil texture, fertilizer management, and cropping systems in controlling N_2_O emissions (Gu et al. 2013; Autret et al. 2019; Ammann et al. 2020). Existing agroecosystem models, which include, among many others, Daycent, DNDC, SWAT, and SPACSYS have specific modules for the quantification of GHG emissions based on varying degrees of process representation (Grosso et al. 2009; Wu et al. 2015; Wagena et al. 2017; Tripathi et al. 2021). However, the application of these physically based data-demanding models to large spatial scales remains challenging because of the difficulty in assembling the required input data to reflect important site-specific parameters. The use of such complex models and proper interpretation of the modeled outputs also require some expert knowledge of the processes and key controls involved. To overcome this complexity, emission factors have been prepared by the IPCC for relevant agricultural activities for national-scale GHG inventory reporting (IPCC 2019b) and country-specific emission factors are being generated to produce smarter inventories for the agricultural sector (e.g., Thorman et al. 2020). These emission factors have been applied at a national scale to map GHG emissions at a 1-km scale in the UK (National Atmospheric Emissions Inventories 2022). These estimates give overall totals in broad categories from all sources, which clearly limit the potential for informing the spatial targeting of mitigation. Equally, grid-scale mapping is very useful for showing generalized spatial patterns but has no direct linkage to the management units used by government policy teams and environmental managers. Consequently, there is an ongoing and important need for evidence-based assessment of the existing status of GHG emissions and projected mitigation potential at appropriate management scales for the development of economically viable, technically feasible, and morally fair strategic pathways for the agricultural sector (Poore and Nemecek 2018; Lynch et al. 2021). Equally, given the need to deliver against various environmental policies, potential co-benefits and trade-offs of mitigation pathways targeting reductions in GHG emissions from agriculture also need to be estimated explicitly.

**Fig. 1:**
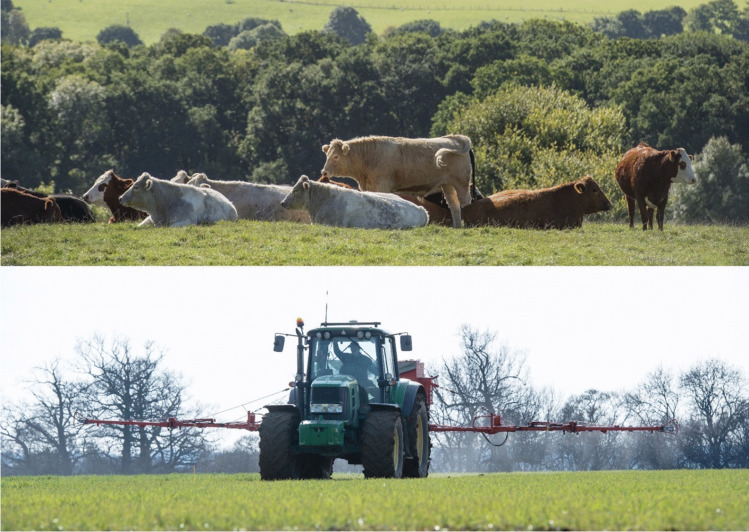
Typical farming activities generating the unintended consequences explored in this study (photos from Rothamsted Research Image Library).

Against this background, this contribution employed a novel farm-based modeling approach to estimate the global warming potentials associated with the business-as-usual (BAU) major farming activities across England (Fig. 1). To account for the differences in the half-life of agricultural-derived GHG in the atmosphere, both GWP20 (representing the average warming potential over a 20-year timeline) and GWP100 (representing the average warming potential over a 100-year timeline) were calculated to account for the distinctive impact of so-called stock pollutants, e.g., nitrous oxide, and flow pollutants, e.g., CH_4_ (Lynch et al. 2021). The former is also more relevant to the UK policy of achieving net zero by 2050. The technical feasibility for the reduction of GWP20 and GWP100 using existing mitigation measures was estimated along with their potential co-benefits for reducing agricultural water pollution and biodiversity loss. The novelty of the work lies in the generation of model farms at a strategic scale using a combination of publicly available and bespoke survey data and importantly, model farms that capture both farm structure (e.g., crop types) and current or potential future uptake of best management practices relevant to farm types.

## The approach

The modeling assessments of GHG emissions under both BAU and a potential alternative management future with increased uptake of on-farm interventions were undertaken using an existing multipollutant modeling framework; namely the Catchment Systems Model (CSM: Zhang et al. 2022; McAuliffe et al. 2022). The full model structure can be visualized in the open-access repository (Collins and Zhang 2024). This framework uses model farms as base units for the quantification of emissions to air and water. For emissions to air, both CH_4_ and nitrous oxide were quantified. Here, the updated IPCC methodology for CH_4_ and N_2_O (IPCC 2019b) with adjustments to the N_2_O calculations to account for improved representation of ammonia (NH_3_) losses based on the National Ammonia Reduction Strategy Evaluation System (NARSES: (Webb and Misselbrook 2004)) was used. Energy use associated with field and farm operations and associated GHG emissions were estimated using the approach reported previously by Gooday et al. (2014). Here, key operations included fertilizer or pesticide applications and manure handling and spreading. The embedded emissions resulting from the production of fertilizers and pesticides were explicitly accounted for, as well as other farming activities, such as storing and drying crops, milking dairy animals, and housing and heating for all livestock types.

**Fig. 2:**
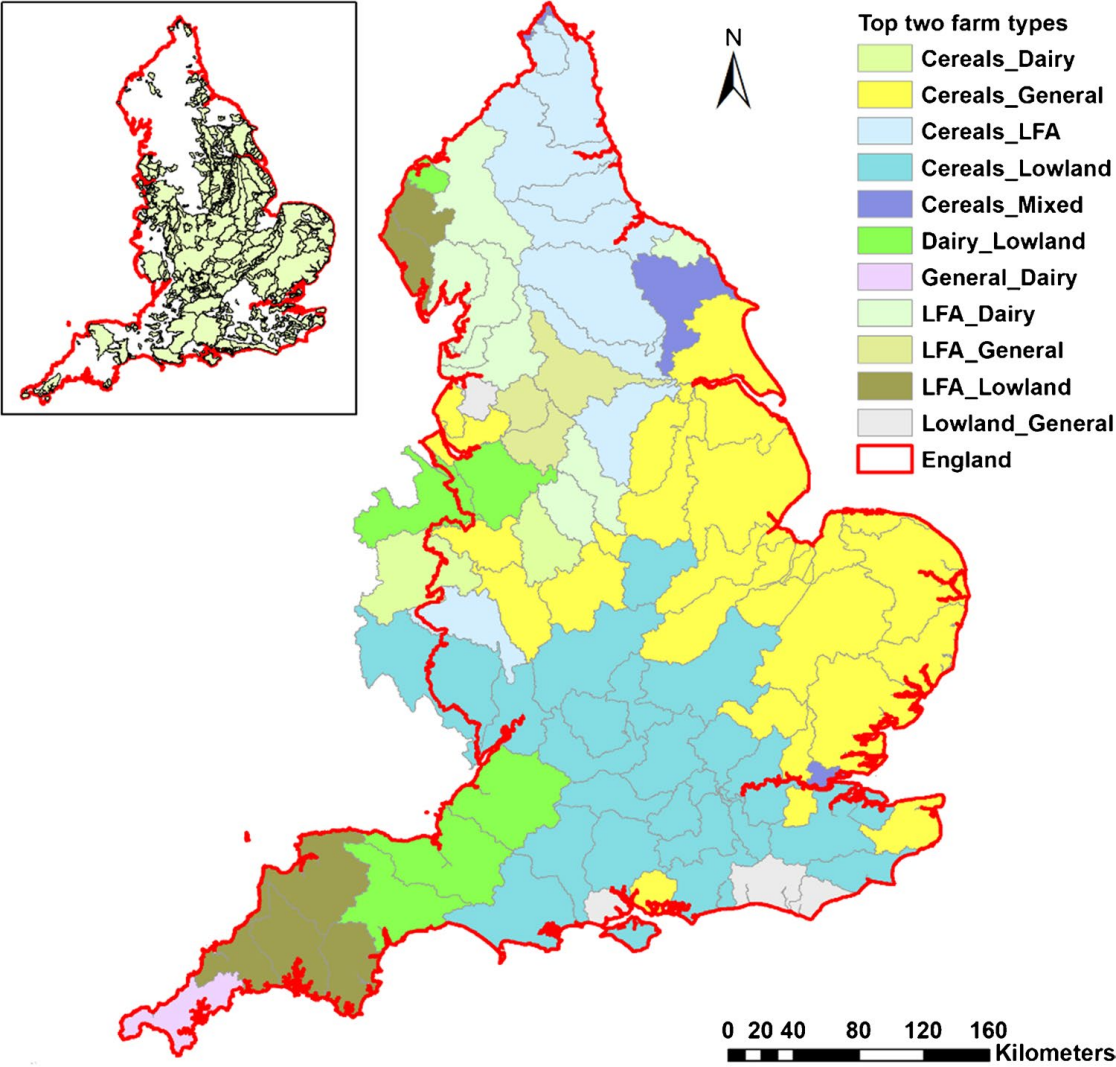
Water management catchments (WMCs) across England and the top two main robust farm types b land areas therein, where “LFA” refers to grazing in less favorable areas, “Lowland” refers to grazing in lowland areas and “General” refers to general cropping. The thumbnail map shows the nitrate vulnerable zones (NVZs).

To support scaling out to estimate agricultural emissions at a broad scale, the so-called water management catchments (WMCs), which lie between Water Framework Directive river basin districts and waterbodies and are used for reporting purposes by UK policy teams, were adopted. The WMCs divide England into 90 spatial units with an average area of ~1500 km^2^, ranging from 105 to over 4000 km^2^ (Fig. 2). For each WMC, multiple model farms were generated to represent the spatial variability of farming activities and their associated impacts on the air and water environments. The construction of model farms was mainly based on the 2019 June Agriculture Survey (JAS) data for England which are grouped on the basis of the robust farm type classification scheme (Defra 2023): cereals, general cropping (hereafter referred to as GC), horticulture, lowland grazing for livestock (hereafter referred to as LGL), LFA (less favored area) grazing livestock (hereafter referred to as LFA), dairy, mixed, specialist pigs (hereafter referred to as pigs), and specialist poultry (hereafter referred to as poultry). Fig. 2 shows the mapped spatial distribution of the two most spatially extensive farm types within each WMC. For WMCs extending into Wales, only data for the utilized agriculture area in England were used. Multiple years (2015–2019) of national average field fertilizer application rates for different crops present in the modeled farm types were estimated based on the British Survey of Fertiliser Practices (Defra 2022) which also provides information about the trend in manure spreading. The spatial patterns of the abiotic environment within each WMC were characterized by two key variables: annual average rainfall and soil drainage status. The former is based on HADUK gridded long-term (1980–2010) annual rainfall data at a 1 km^2^ scale (Met Office et al. 2018). The soil drainage status is based on derived drainage classes (free draining, drained for arable, and drained for arable and grass) assigned to soil series mapped in the NATMAP1000 vector data product (National Soil Resources Institute, Cranfield University, UK). The registered business addresses of the farms which participated in the 2019 JAS were mapped in each WMC. Unique combinations of robust farm types and their associated intrinsic environment conditions (i.e., rainfall, soils) were identified and treated as representative model farms for each WMC. Farm-type specific GHG emissions, plus emissions to water, were then evaluated for two scenarios. The first represented BAU which includes the impacts of farm structure (i.e., crop areas, animal types, numbers, and ages) and the current uptake of best management measures due to regulation, incentivization including agri-environment schemes, and on-farm advice. The second scenario represented the maximum technically feasible impacts resulting from full (i.e., increased uptake where current implementation rates leave gaps) implementation of all available best management measures driven by the combination of regulation, incentivization, and advice. The mitigation measures with considerable existing uptake (>5%) are listed in Tables 1 and 2, and their existing uptake rates were based on the Defra Farm Practice Survey on GHG mitigation in 2019 (Defra 2019). The full measures considered are shown in Table S1.

**Table 1:** List of on-farm measures included for the modeling of the maximum technically feasible mitigation scenario with ranges in prior uptake rates (%) among modeled farms.

Measures	Minimum	Maximum
Cultivate land for crops in spring rather than autumn, retaining over-winter stubbles	2	80
Reduce dietary N and P intakes: dairy, pigs, poultry	10	80
Do not apply manufactured fertilizer to high-risk areas	25	80
Fertilizer spreader calibration	25	80
Integrate fertilizer and manure nutrient supply	25	80
Do not apply manure to high-risk areas	50	100
Site solid manure heaps away from watercourses/field drains	50	100
Use a fertilizer recommendation system	50	100
Adopt reduced cultivation systems	2	50
Manure spreader calibration	10	50
Capture of dirty water in a dirty water store	50	80
Treatment of PPP washings through disposal, activated carbon, or biobeds	50	80
Cultivate compacted tillage soils	25	50
Farm track management	25	50
Fence off rivers and streams from livestock	25	50
Use correctly inflated low-ground pressure tires on machinery	25	50
Establish cover crops in the autumn	2	25
Establish riparian buffer strips	10	25
Incorporate manure into the soil	10	25
Leave autumn seedbeds rough	10	25
Manage over-winter tramlines	10	25
Minimize the volume of dirty water produced	10	25
Ditch management	0	50
Use slurry band spreading application techniques	2	10

**Table 2:** List of on-farm measures included for the modeling of the maximum technically feasible mitigation scenario without ranges prior uptake rates (%) among modeled farms.

Measures	Rate
Adopt phase feeding of livestock: dairy, pigs	80
Allow cattle slurry stores to develop a natural crust	80
Construct bridges for livestock crossing rivers/streams	80
Reduce field stocking rates when soils are wet	80
Early harvesting and establishment of crops in the autumn	50
Loosen compacted soil layers in grassland fields	50
Move feeders at regular intervals	50
Cultivate and drill across the slope	25
Re-site gateways away from high-risk areas	25
Washing down of dairy cow collecting yards	25
Additional targeted bedding for straw-bedded cattle housing	10
Establish in-field grass buffer strips	10
Extend the grazing season for cattle	10
Improved livestock through breeding	10
Increase scraping frequency in dairy cow cubicle housing	10
In-house poultry manure drying	10
Install covers to slurry stores	10
Locate out-wintered stock away from watercourses	10
Reduce the length of the grazing day/grazing season	10
Use clover in place of fertilizer nitrogen	10
Use high-sugar grasses	10
Use manufactured fertilizer placement technologies	10
Beetle banks	2
Compost solid manure	2
Construct troughs with concrete base	2
Cover solid manure stores with sheeting	2
Establish new hedges	2
Frequent removal of slurry from beneath-slat storage in pig housing	2
Leave residual levels of non-aggressive weeds in crops	2
Management of arable field corners	2
Management of grassland field corners	2
Management of in-field ponds	2
Management of woodland edges	2
Plant areas of the farm with wild bird seed/nectar flower mixtures	2
Skylark plots	2
Uncropped cultivated areas	2
Uncropped cultivated margins	2
Undersown spring cereals	2
Unfertilised cereal headlands	2
Unharvested cereal headlands	2
Use liquid/solid manure separation techniques	2

In addition to representing farm structure (i.e., cropping areas and types, livestock types and ages), CSM also includes explicit representation of on-farm best management practices for soils, manures, fertilizers, pesticides, animals, and farm equipment and infrastructure (Zhang et al. 2022). The uptake rates under BAU were based on previous policy-focused work (Zhang et al. 2017) but where relevant, adjusted using the data reported in Defra farm practices surveys (e.g., Defra 2019) and agri-environment scheme information (i.e., Natural England (2016)). Here, the efficacy assigned to each individual on-farm measure is based on a combination of experimental evidence and elicitation of expert opinion (e.g., Cuttle et al. 2016). The list of mitigation measures included in the GHG mitigation scenario is provided in Tables 1 and 2. CSM assumes that the interactions between on-farm measures are multiplicative, rather than additive, to avoid over-estimation of impacts as shown below, where *E*_*t*_ is the overall reduction in %, *E*_*i*_ is the % reduction for individual measures concerned, and *n* is the number of measures.

$${E}_{t}=100- \prod_{i=1}^{n}\left(100- {E}_{i}\right)$$

Recommended conversion coefficients in IPCC reports (Smith et al. 2021) were used to estimate GWP20 and GWP100 from the modeled CH_4_ and N_2_O annual loads. While a single value of 273 was used for the N_2_O conversion, two different values were used for CH_4_: 81.2 for GWP20 and 27.9 for GWP100, respectively.

The total GWP20, GWP100, and other quantitative assessments (e.g., nitrate, phosphorus, and sediment loads to water) for each WMC were calculated as the multiplication of model farm-based estimates with the corresponding holding counts. These totals were further normalized by utilizing agricultural areas to permit direct inter-WMC comparisons. Because of the non-normal distributions of the estimated GHG emissions, a non-parametric approach was used to calculate the coefficient of variation (CV), viz.:

$$CV=\frac{{P}_{95} -{P}_{5}}{median}*100$$

where P_5_ and P_95_ are the 5^th^ and 95^th^ percentiles of the sample population, respectively.

The potential benefit of on-farm best management practices for terrestrial biodiversity is based on the impacts of agri-environment measures on key taxonomic groups comprising plants, invertebrates, and birds, as summarized in Boatman et al. (2008; 2010). The latter reviewed specific studies on species within the individual taxonomic groups, including, for example, bryophytes (Bosanquet 2003) for plants, spiders, and carabid beetles (Hassall et al. 1992) for invertebrates and the stone curlew and cirl bunting for birds (Grice et al. 2007). CSM computes the impacts of best management practices on biodiversity using an index score, rather than quantitative units. The higher the positive score, the more positive the impact on biodiversity.

## Results and discussion

### Spatial pattern of farm types across England

The farm types included in the modeling occupy about 90,324 km^2^ of land, accounting for around 69% of the physical area of England. Cereal farming is the most extensive land use (~33%), followed by GC, LGL, and LFA grazing (~15% each). Specialized farm types, including horticulture, pigs, and poultry use the least amount (< 2%), and dairy and mixed are both~10%. As expected, the locations of these different farm systems manifest a strong regional variation (Fig. 2). Annual rainfall is one of the key controlling variables for the spatial distribution of the different farm types since there is an upper limit of around 900 mm for cereal farms and 700 mm for LFA farms. Dairy farms have a wider spatial distribution than the other farm types. As for soil drainage status, cereal farms can be found in all types of soils in roughly equal proportions. All livestock farms, including dairy, LGL, and LFA grazing tend to be less common on either free-draining land or land drained for arable and grassland use. The other farm types, such as horticulture, mixed, and GC are more likely to be on free-draining soils.

An important policy instrument for farming in England concerns the EU Nitrates Directive 91/676/EEC) which was introduced in 1991 to protect water quality from pollution by agricultural sources. This instrument has been used to designate so-called nitrate vulnerable zones (NVZs) which cover ~55% of land in England and which were last reviewed in December 2020. Farms in NVZs must adhere to manure and fertilizer storage, handling, and application rules. The spatial distribution of farm types in Fig. 2 indicates that more cereal farms (~79%) than any other farm type are located in the designated NVZ area. In comparison, only 7% of LFA grazing farms are in NVZ areas. These spatial patterns are important since the enforcement of NVZ-related measures is expected to affect GHG emissions as nitrogen fertilizer use is known to be a key source of soil-related N_2_O emissions.

### Comparison of modeled methane and nitrous oxide emissions against reported GHG inventories

Modeled CH_4_ and N_2_O emissions for each WMC were compared against the reported 2019 inventories (National Atmospheric Emissions Inventory) for the corresponding area, where relevant gridded outputs at 1 km × 1 km resolution were used. The scatter plots of WMC scale averages from the two approaches are shown in Fig. S1. For both gases considered, strong linear correlations were found with the corresponding r^2^ at 0.91 and 0.78 for methane and N_2_O, respectively. These results suggest that the N_2_O data exhibit greater differences, especially in the case of high-emission areas. Regardless, the evaluation using the national inventory data suggests that the modeled outputs can underestimate N_2_O emissions***.***

The observed agreements for CH_4_ emissions could be explained by the common livestock information embedded in the national census data and the application of the same IPCC methodology. The differences in N_2_O could be attributed to the different approaches adopted and the parameterization of the key inputs, e.g., fertilizer application rates. Similar results were reported by previous work (Zhang et al. 2017) where the evaluations were undertaken at a coarser scale, i.e., using river basin districts rather than WMCs. There are few comparable studies at such a scale. One related work is the estimation of farm-level GHG emissions in Scotland (c.f., Scottish Government 2023) where a similar ranking of GWP100 among comparable farm types has been reported, but with higher absolute magnitudes, ranging from 2.7 to 17.2 t CO_2_ eq ha^−1^ year.

### Spatial variability of estimated GWP20 and GWP100 at farm scale

The quantification of GHG emissions is the foundation of GWP estimation. Table 3 presents summary statistics for the estimated annual specific emissions of CO_2_, CH_4_, and N_2_O at the farm scale. The overall rankings of the specific loadings for the modeled farm types are as follows: pigs > poultry > dairy > mixed > cereals > LGL > GC and horticultural > LFA for N_2_O, compared with dairy > LGL > mixed > LFA > pigs > poultry > cereals > GC/horticulture for methane, and dairy > cereals > mixed > GC > poultry and horticultural > LGL> pigs > LFA for CO_2_, respectively. Relatively speaking, the differences among farm types are largest for CH_4_ and smallest for CO_2_ emissions associated with on-farm energy use. This confirms the unique contribution of methane emissions from livestock. Significant linear relationships (r^2^>0.8) were found between the emissions of N_2_O and CO_2_ for some farm types (LFA, LGL, GC, horticulture). The relationships for the cereal, dairy, and mixed farms showed much greater scatter (r^2^ < 0.6). For those farm types with significant indoor operations, i.e., pigs and poultry, no linear relationships were found.

**Table 3:** Estimated specific annual loadings (kg ha^−1^) of nitrous oxide, methane, and carbon dioxide for the model farm types across England. CO_2_ eq. is associated with energy use on farms only and excludes embedded emissions. P_5_ is the 5th percentile. P_95_ is the 95th percentile. *GC*, general cropping; *LFA*, less favorable area; *LGL*, lowland grazing livestock.

Farm types	CO_2_ eq.			CH_4_			N_2_O			Sample
	P_5_	median	P_95_	P_5_	median	P_95_	P_5_	median	P_95_	counts
Cereals	982	1223	1378	1.7	3.7	7.3	2.15	2.63	3.16	923
Dairy	1331	1575	1802	190.2	288.8	392.5	3.93	5.5	7.05	635
GC	684	943	1339	0	0	0	1.28	1.82	2.71	1002
Horticulture	731	875	1138	0	0	0	1.35	1.8	2.38	648
LFA	289	380	458	39.5	55.5	81.3	0.97	1.31	1.8	455
LGL	484	593	714	51.8	99.5	147.5	1.43	2.32	3.16	1048
Mixed	812	1024	1234	45.3	76.9	104.4	2.55	3.4	4.23	837
Pigs	111	439	1786	3	14.7	218	4.3	9.6	19.8	809
Poultry	190	814	2105	2.3	12.2	197.3	4	8.8	23.6	639

The pollutant types, their magnitudes, and variability across the country are clearly dependent on farm type and the corresponding intensity of management. For CH_4_, insignificant emissions are expected from arable farms given the general absence of animals. In contrast, the high stocking densities and intensive management on dairy farms make them distinctive from all other farm types in that they generate the highest specific loadings of all three gases considered herein. With an overall national median annual specific CH_4_ emission of 289 kg ha^−1^, dairy farms are responsible for losses of this pollutant to the atmosphere that are nearly 3 times the corresponding second-highest loading which is from LGL grazing farms (Table 3). In the case of N_2_O, the overall national median annual specific loading from dairy farms is still ~60% higher than that from mixed farms. Excluding off-farm embedded emissions, dairy farms were predicted to release ~30% more CO_2_ from on-farm energy use than the other farm types. However, in the case of CO_2_ emissions, the between model-farm variations are relatively smaller in comparison with those for CH_4_. LFA grazing farms were predicted to generate the lowest specific annual loadings of CO_2_. Overall, pigs and poultry farms exhibit much higher between model-farm variability (>50%), especially for CH_4_ (>128%). For the other farm types, the estimated coefficients of variation are mostly <30% (see Table 4).

**Table 4:** Estimated coefficient of variation (%) for specific loadings across different WMCs. No embedded emissions were considered for CO_2_ eq., GWP20 and GWP100. *GC*, general cropping; *LFA*, less favorable area; *LGL*, lowland grazing livestock.

Farm types	CO_2_ eq.	CH_4_	N_2_O	GWP20	GWP100
Cereals	10.1	45.2	11.6	8.2	9.1
Dairy	17.9	20.4	17.2	19.4	18.0
GC	29.5	NA	23.4	21.7	21.7
Horticulture	27.4	NA	17.3	14.6	14.6
LFA	19.1	24.3	20	23.2	21.8
LGL	21.4	29	24.1	27.5	25.5
Mixed	14.5	25.2	15.6	20.3	16.1
Pigs	99.9	128.7	50.1	64.4	50.8
Poultry	66.5	131.2	57.6	40.4	39.7

**Fig. 3:**
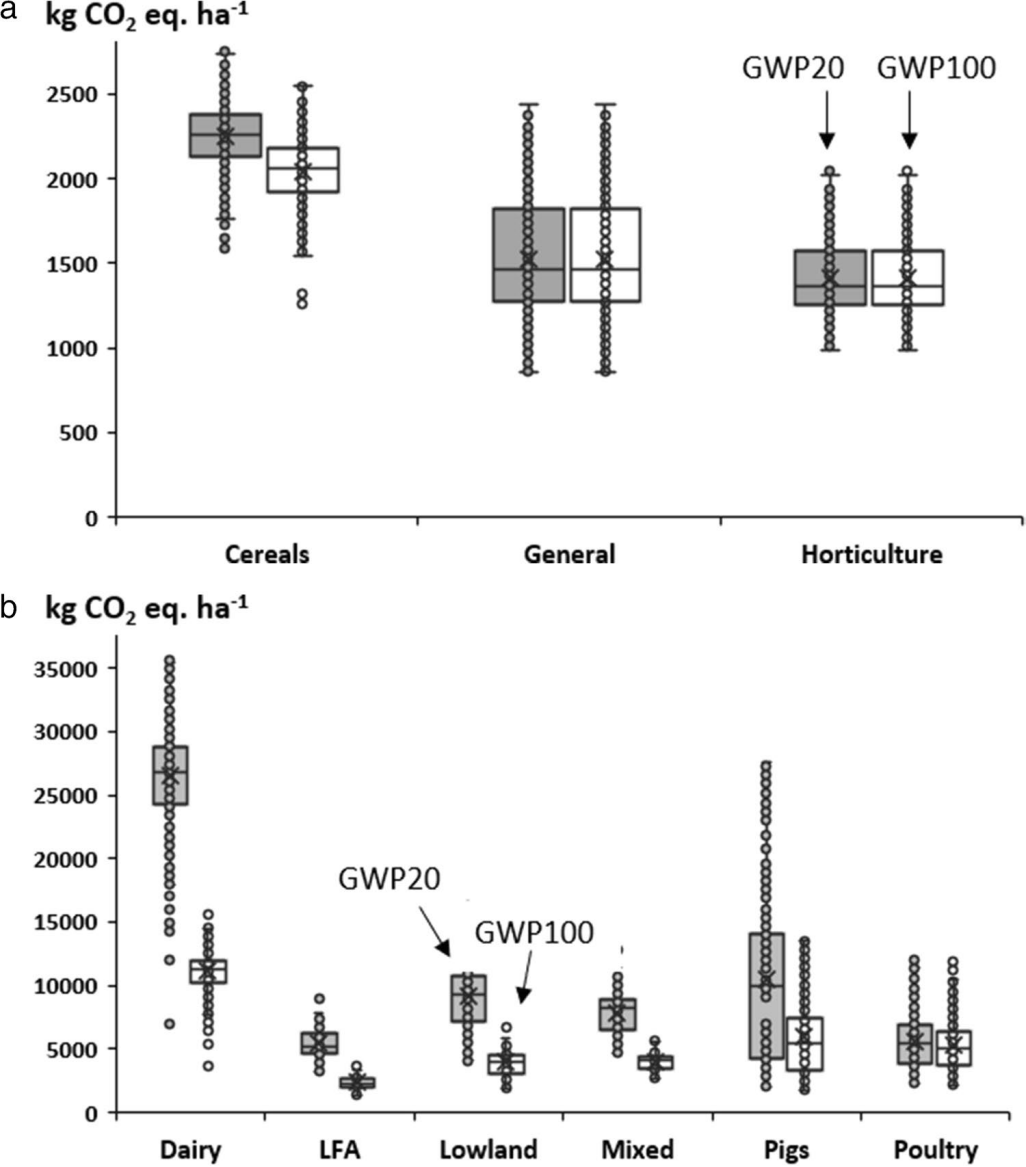
Estimated farm scale annual GWP20 and GWP100 for farms without (**a**) and with (**b**) livestock.

The estimated annual GWP20 and GWP100 at the farm scale are shown in Fig. 3. For farm types without livestock (Fig. 3a), the average values of GWP20 and GWP100 were predicted to be <1500 kg CO_2_ eq ha^−1^ and <1200 CO_2_ eq ha^−1^, respectively. Given the low CH_4_ contributions for these farm types, the differences between GWP20 and GWP100 are small. For the farm types with livestock, the predicted GWP20 varied between 5305 kg CO_2_ eq ha^−1^ and 25,775 kg CO_2_ eq ha^−1^ for LFA and dairy farms (Fig. 3b). For comparison, mixed and LGL farms were predicted to have corresponding average values of 7318 kg CO_2_ eq ha^−1^ and 8886 kg CO_2_ eq ha^−1^, respectively. The differences between GWP20 and GWP100 for this group of farm types are clearly greater, with the average values for the former all being more than double those for the latter (Fig. 3b). Among the individual farm types, cereal, dairy, and horticulture exhibited smaller spatial variations in GWP20 and GWP100, with estimated coefficients of variation being <20%. Again, the specialized farms, i.e., pig and poultry, exhibited much higher (40–64%) variation among the model farms across the country (Table 4).

The results discussed so far have not considered the embedded GHG emissions associated with the use of fertilizers and pesticides. Their significant contributions to GWP20 and GWP100, especially in the case of arable farms, can be seen in Table 5. With embedded emissions included, CO_2_ becomes the dominant gas for GWP20 and GWP100, accounting for >50% in the case of all non-specialized farm types without livestock. If the embedded emissions are excluded, N_2_O becomes the dominant gas accounting for GWP20 and GWP100 and CO_2_ becomes secondary for some farm types. In contrast, for the non-specialized farm types with livestock, CH_4_ is the overwhelming gas accounting for GWP20 and GWP100 (Table 5). Its relative contributions are >70% for GWP20 and >50% for GWP100 under both assumptions concerning embedded emissions. Within this group of farm types, the relative contribution of CH_4_ to GWP20 and GWP100 is highest for dairy and lowest for mixed farms (Table 5). For the specialist farm types, i.e., pigs and poultry, N_2_O dominates both GWP20 and GWP100, but with a more significant contribution when embedded emissions are excluded.

**Table 5:** Percentage contributions of different greenhouse gases (GHG) sources to GWP20 and GWP100 at farm scale: with and without embedded emissions. *GC*, general cropping; *LFA*, less favorable area; *LGL*, lowland grazing livestock.

GHG emission indicator	Farm type	With embedded emission			Without embedded emission		
		CO_2_ eq.	CH_4_	N_2_O	CO_2_ eq.	CH_4_	N_2_O
GWP20	Cereals	53.7	14.3	32.1	24.7	22.8	52.6
	Dairy	6.1	88.2	5.7	3.3	90.8	5.9
	GC	65.8	0.0	34.2	39.7	0.0	60.3
	Horticulture	64.9	0.0	35.1	33.6	0.0	66.4
	LFA	6.8	86.5	6.7	2.9	90.1	7.0
	LGL	6.7	86.3	6.9	3.3	89.5	7.2
	Mixed	13.5	74.6	11.9	5.7	81.2	13.0
	Pigs	23.6	34.8	41.7	11.6	38.9	49.5
	Poultry	26.4	32.6	41.0	17.4	35.6	47.0
GWP100	Cereals	59.1	5.5	35.4	28.9	9.5	61.6
	Dairy	14.5	72.0	13.5	8.2	77.3	14.5
	GC	65.8	0.0	34.2	39.7	0.0	60.3
	Horticulture	64.9	0.0	35.1	33.6	0.0	66.4
	LFA	15.6	68.9	15.5	7.0	75.9	17.1
	LGL	15.4	68.6	16.0	8.0	74.6	17.4
	Mixed	26.1	50.6	23.2	12.2	60.1	27.7
	Pigs	28.6	20.2	51.2	14.5	23.4	62.0
	Poultry	31.7	18.5	49.7	21.3	20.8	57.8

### Spatial variability of estimated GWP20 and GWP100 at WMC scale

**Fig. 4:**
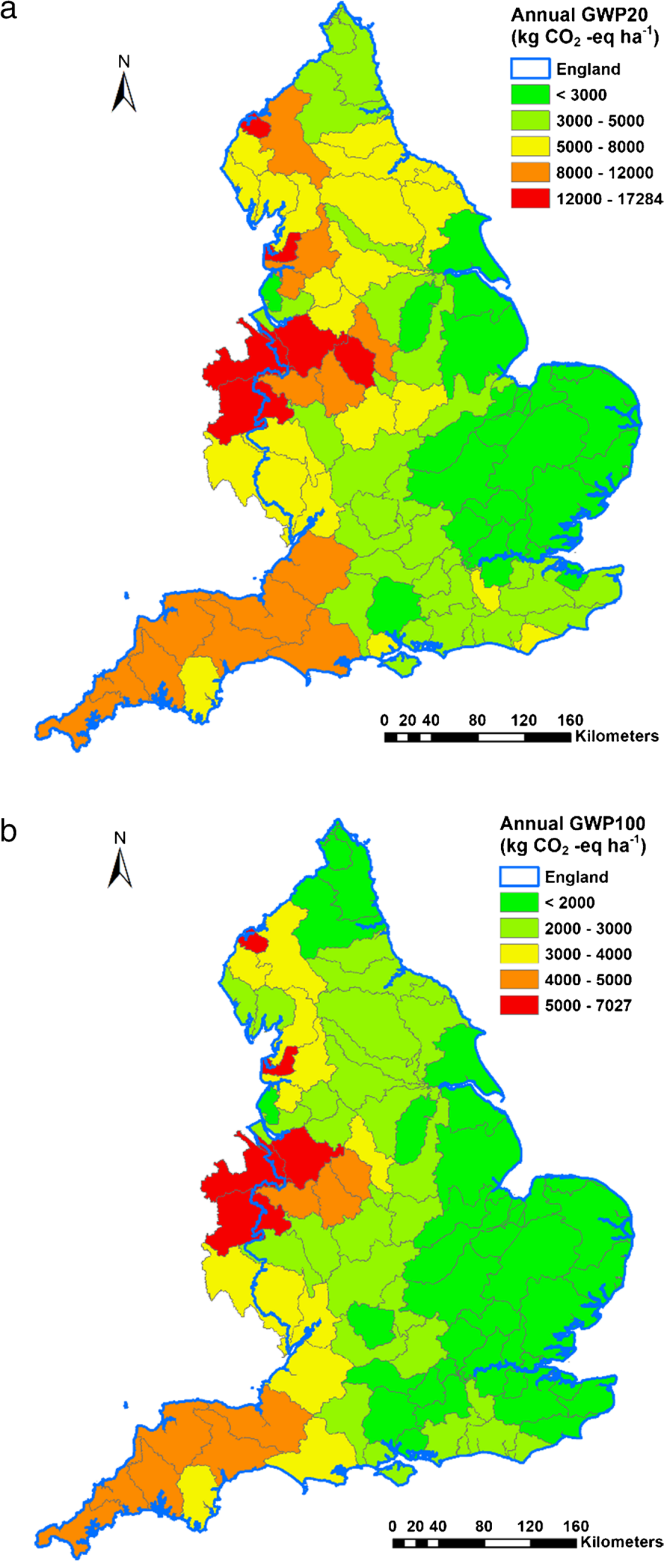
Mapped spatial patterns of GWP20 and GWP100.

The total annual agricultural gaseous emissions for any given WMC across England depend on the abiotic environment and farm type composition. Fig. 4 presents maps of annual GWP20 and GWP100 (excluding embedded emissions) from agriculture across England at the WMC scale, wherein the gaseous loadings were scaled by corresponding farmed areas. For England as a whole, the median GWP20 and GWP100 were estimated to be 4606 kg CO_2_ eq. ha^−1^ and 2334 kg CO_2_ eq. ha^−1^. Though there are some exceptions, the overall patterns suggest an east-west split wherein the former has much lower gaseous emissions. The contribution of CH_4_ from livestock grazing is one important driver for this regional contrast. It is also clear that the differences between the WMCs are greater for GWP20, with the inter-quartile range estimated to be 4240 kg CO_2_ eq. ha^−1^. The corresponding interquartile range for GWP100 is estimated at 1462 kg CO_2_ eq. ha^−1^. Corresponding coefficients of variation can be as high as 57% for GWP20 and 47% for GWP100. The inclusion of embedded emissions from agrochemical use on farms increases the magnitude of the mapped specific gaseous loadings constituting GWP20 and GWP100.

**Fig. 5:**
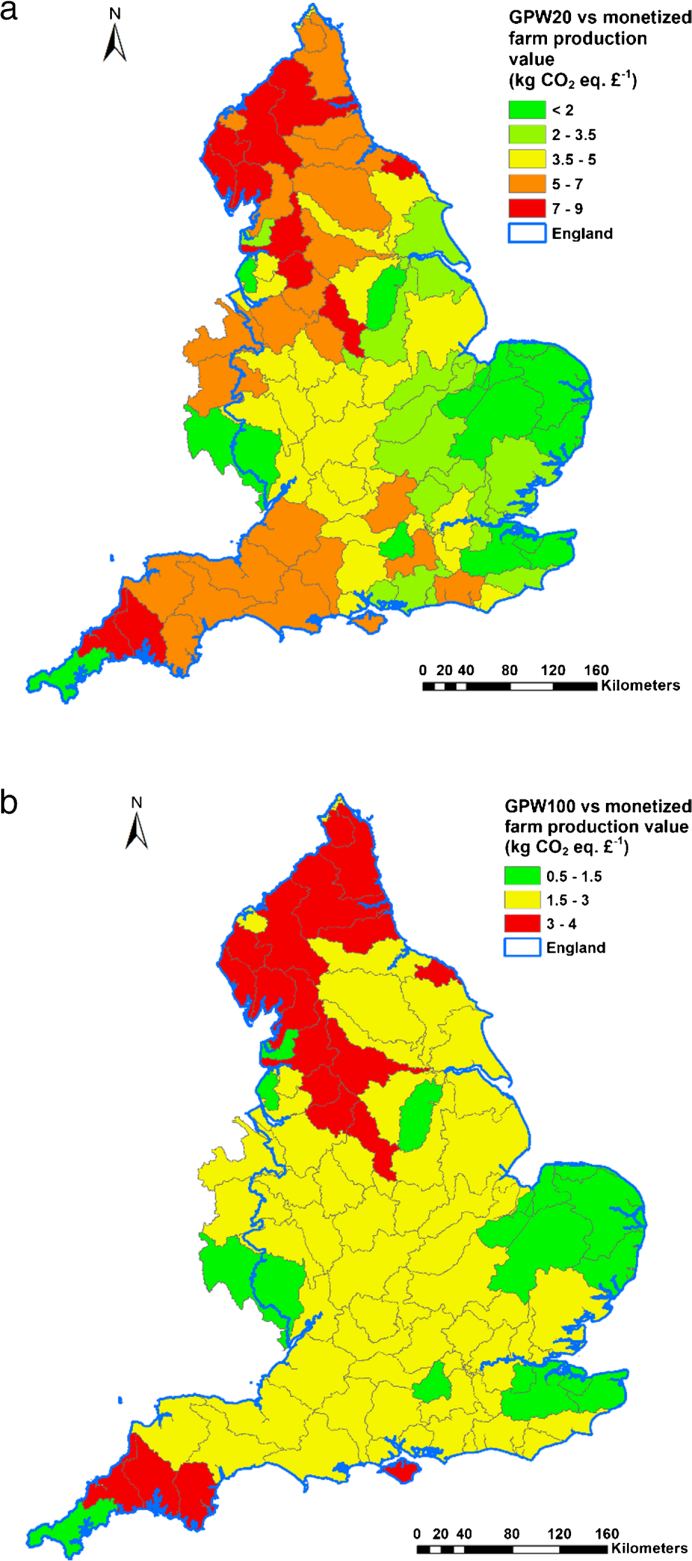
Estimated ratios of GWP20 (**a**) and GWP100 (**b**) against farm total production values.

GHG emissions represent one important unintended environmental consequence of BAU farming. Here, it is useful to gauge the spatial variation of environmental damage costs associated with agricultural atmospheric emissions represented by GWP20 and GWP100 against the economic benefits generated by monetized farm production (Fig. 5). Defra-recommended carbon values for 2020 have a median value of £241 (ranging between £123 to £336). The estimated ratios for GWP20 range from 0.58 to 8.89 kg CO_2_ eq. £^−1^ farm production, with an overall national average of 4.2 kg CO_2_ eq. £^−1^ farm production. This means that for every ton of equivalent carbon emitted, the corresponding production value is around £238. The corresponding ratios for GWP100 exhibit a narrower range (0.53 to 3.99 kg CO_2_ eq. £^−1^ farm production) and a lower national average value (2.35 kg CO_2_ eq. £^−1^ farm production). This indicates that the carbon value only represents the emission potential in the near future, and it could increase significantly (around £416) if the long-term emission potential, as indicated by GWP100, is considered. With the current work herein, the nutrient contents of farm production are not explicitly considered. The ratio of carbon emissions potential to economic and human health benefits could change if their spatial variations are considered explicitly.

### Mitigation of GHG emissions through on-farm management and associated co-benefits for water pollution and terrestrial biodiversity

**Fig. 6:**
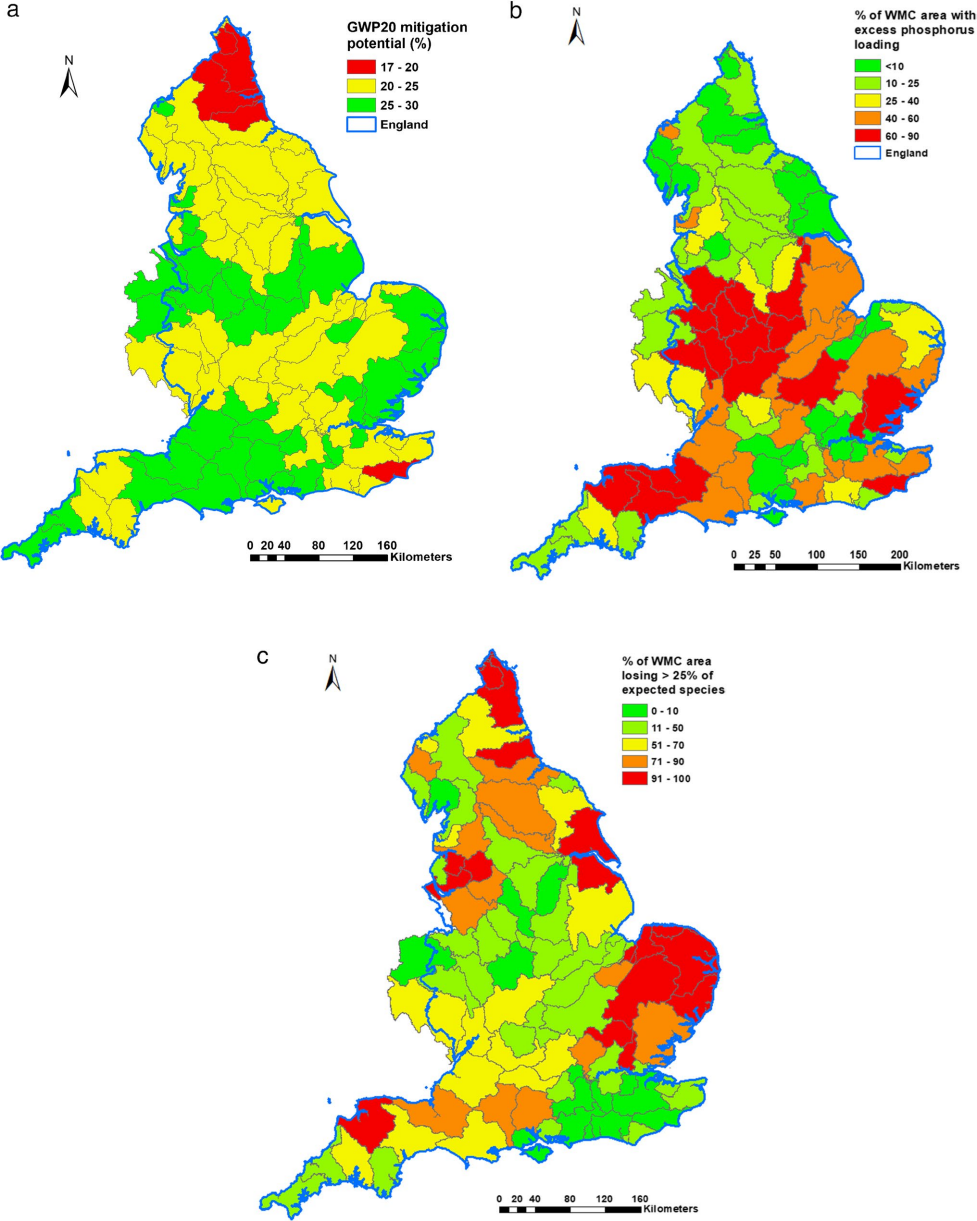
Mapped maximum technical feasibility for the mitigation of GWP20 (**a**), spatial distribution of excess phosphorus loadings (**b**), and the loss of a quarter of native species (**c**) at WMC scale.

Assuming no change in farm structure (e.g., changes in land cover or BAU animal stocking densities) and no economic constraints to the implementation of on-farm best management measures for controlling GHG emissions, the technically feasible maximum mitigation potential for both GWP20 and GWP100 associated with better farm management was evaluated using the full implementation of all available measures on all farm types in each WMC and the spatial pattern for the former is shown in Fig. 6a. The spatial pattern for the latter is provided in Fig. S2. There are slight differences in the spatial patterns for the two time periods considered (i.e., 20 years vs 100 years). However, both have similar ranges of variation (17–30% for GWP20 and 19–27% for GWP100) and median values (~24%) for the technically feasible maximum mitigation potential. Clearly, on this basis, improved farm management alone, without structural change, will not be able to achieve the net zero policy goal. The modeled values for the mitigation potentials for GHG emissions and GWP20 or GWP100 only represent what is technically feasible without considering many other constraints, including financial feasibility and the practicability of integration into existing farming operations associated with any given farm system type.

For policy support purposes, it is informative to assess if the same GHG measures could contribute to the improvement of other ecosystem services, including, for example, water quality regulation. Based on a strategic assessment in 2019, there are 418 and 1469 Water Framework Directive waterbodies failing to achieve “good ecological status” due to excess sediment and phosphorus loadings, respectively (Environmental Agency 2018/). The spatial distribution in terms of the WMC spatial units used in this study for phosphorus is shown in Fig. 6b and for sediment in Fig. S3. Compared with Fig. 6a, it is clear that there is an opportunity to explore the scope for delivering some co-benefits from interventions selected principally for reducing GHG emissions, especially in the midland and eastern areas of the country, and especially for simultaneous reductions of GWP20 and phosphorus emissions to water. CSM was therefore used to estimate the magnitude of co-benefits for water pollutant reductions. It is estimated that the magnitude of co-benefits has a similar median value of ~34% for both sediment (39 WMCs affected) and phosphorus (69 WMCs affected), but the sediment reductions exhibit higher variability with a CV of 30%, compared with 14% for phosphorus. The similarity of the technically feasible mitigation efficacies for co-benefits associated with sediment and phosphorus reductions could be due to the dominance of the particulate form of phosphorus and the significant impacts of the on-farm measures selected for GHG reduction on soil management. Strong co-benefits for water quality could be expected to arise from the implementation of on-farm measures for the reduction of GHG emissions as both outcomes share some similar pollutant sources, mobilization processes, and delivery pathways on agricultural land.

Dyer and Oliver (2016) mapped the ecological status of the UK at a 10 km^2^ grid scale and developed a biodiversity indicator wherein surveyed species were compared against the expected species for various landscapes (Dyer et al. 2016). The mapped indicators, expressed as ratios, were summarized for each WMC and mapped (Fig. 6c) where the proportion of the total area of each individual WMC which has lost >25% of native species was depicted. Comparing this map with Fig. 6a, it is possible to identify areas to assess whether the improved mitigation of GHG emissions might also deliver co-benefits for biodiversity. While the quantification of any specific co-benefits for biodiversity remains a challenge, examination of the mitigation efficacy of those on-farm measures included in the GHG mitigation scenario (Tables 1 and 2) that are also known to deliver benefits for terrestrial biodiversity suggests that several individual options with known effects for the reduction of N_2_O emissions and farm energy use could also enhance the biodiversity scores of farmlands (Table 6).

**Table 6:** Selected on-farm best management measures which could potentially reduce GHG emissions (%) and deliver co-benefits for terrestrial biodiversity (scores). Only N_2_O is used to represent GHG emissions here, since no positive effects of CH_4_ mitigation for biodiversity are included in the CSM modeling framework.

Description of mitigation measure	N_2_O emission reductions	Energy use reductions	Biodiversity
Management of in-field ponds	−10		5
Uncropped cultivated areas	−10	−10	5
Undersown spring cereals	−50	−50	2.5
Cultivate land for crops in spring, retaining over-winter stubbles	−10		2.5
Establish and maintain artificial wetlands-steading runoff	−25		1
Use clover in place of fertilizer nitrogen	−10	−40	1
Establish cover crops in the autumn	−50	75	0.2
Early harvesting and establishment of crops in the autumn	−25		0.2
Adopt reduced cultivation systems	−10	−50 to 25	0.2
Leave residual levels of non-aggressive weeds in crops		−10	2.5

While the inclusion of biodiversity in our work considered key taxonomic groups comprising plants, invertebrates, and birds, there is a growing body of evidence that healthy soils are a fundamental requirement for the effective functioning of agroecosystems and the delivery of goods and services (Dominati et al. 2010; Baveye et al. 2016). In particular, healthy soils accommodate diverse assemblages of organisms (Fierer et al. 2009). Rutgers et al. (2019) used a proxy indicator system for modeling and mapping soil biodiversity in European soils based on biological and chemical attributes shortlisted in work reported by van Leeuwen et al. (2017). Overall, soil biodiversity was shown to be higher in grassland than in arable soils (Tsiafouli et al. 2015). On this basis, the need for improving soil biodiversity would be greater in the east of England, compared to the west, and would therefore generally agree, with the spatial targets (Fig. 6c) identified for biodiversity using wider taxonomic groups identified by Dyer and Oliver (2016).

### Modeling limitations

While efforts were made to represent the different management practices, such as fertilizer use and manure spreading, associated with distinctive farming types based on national surveys, potential regional variations resulting from WMC catchment-specific environmental conditions and mitigation efforts were still not fully accounted for. Little data are available concerning the movement of manures among farms, including import and export, which could have some implications for the mapped patterns if a catchment has a small area but with a significant presence of specialized livestock farms, such as poultry or pig farms. However, given the median WMC area of >1300 km^2^, the overall impacts of these types of limitations will be small.

For the modeling of mitigation impacts, typical efficacy estimates were used, based on a mix of experimental evidence and expert opinion. No attempts were made to incorporate the ranges of efficacy that could be expected due to a range of factors including, for example, farm-to-farm variations in the maintenance or spatial targeting of a specific mitigation measure. As a result, the mapped spatial patterns only represent the predicted average outcomes which could have varying degrees of uncertainty, depending on the details surrounding applicable mitigation methods for any individual catchment. Another key area of uncertainty concerns the assumed interactions between the individual on-farm interventions. For simplicity and to avoid over-estimation of impacts, a multiplicative approach is used, but in reality, interactions between some interventions could be more additive. Current empirical work tends to focus on the assessment of individual interventions, as opposed to combinations thereof, and even the former is commonly limited to specific geographical contexts driven by the locations of experimental platforms rather than being structured to provide truly strategic data representative of variation in the physical environment. Explicit uncertainty analysis would be necessary to help address some of the above limitations, wherein optimization of measure selection for individual catchments is required. This study has estimated both GWP20 and GWP100 to demonstrate the warming effects of GHG, especially methane, over different timespans. It is recognized, however, that alternative methods, such as GWP* (Lynch, et al. 2020), are available.

### Policy implications

So-called GHG values or “carbon values” are used across the UK government for valuing GHG emissions and any changes thereof resulting from intervention strategies. These values provide monetization that society places on one ton of CO_2_ equivalent (£/t CO2 eq.). Importantly, carbon values differ from carbon prices, which represent the observed price of carbon in a relevant market (such as the UK Emissions Trading Scheme). To help guide the delivery of the UK legal target of net zero by 2050, the UK calculates 5-yearly carbon budgets, and these are based, in part, on the application of annual carbon prices which are based on a target-based approach or marginal abatement costs rather than the social costs of carbon (UK Department of Energy and Climate Change 2009). Published carbon values for 2020 (i.e., the closest published values with land use data used for modeling in the study reported herein) comprise a central series of 241 £/t CO_2_ eq., with a corresponding low and high series of 120–361 £/t CO_2_ eq. The full range reflects a plus or minus 50% sensitivity about the central series. Combining these values with the estimated national average GWP20 of 4.2 kg CO_2_ eq. £^−1^ farm production, under BAU, suggests that the typical carbon values for farm production range between £ 0.50 and 1.51/£ farm production, with a corresponding average of £1.01/£ farm production. Taking account of the predicted technically feasible national average impact (~24% reduction) of on-farm GHG mitigation on GWP20 generates equivalent estimates of £ 0.38–1.15/£ farm production, with a corresponding average of £0.77/£ farm production.

With regards to delivering co-benefits from the drive for transitioning towards net zero, Table 6 provides a shortlist of on-farm interventions to inform stakeholders. Focusing more on net zero alone, the modeled mitigation scenario points very clearly to the need for structural land cover change on farms for delivering net zero in agriculture across England, since the full uptake of a long list of on-farm mitigation measures (Tables 1 and 2) for GHG management delivered only a reasonably limited (median ~24%) reduction in GHG emissions. To support the implementation of land use change for net zero, UK science funding is currently supporting demonstrators for GHG reduction (GGR) technologies comprising enhanced rock weathering, biochar, perennial biomass crops, woodland creation and management, and peatland restoration. Collectively, these demonstrators will provide fundamental evidence required to support farmers in decision-making for progressing towards net zero.

## Conclusions

While exploring and implementing scenarios for delivering net zero remains a policy priority in England, and indeed, many nations worldwide, it is vitally important to understand any potential co-benefits for wider policy objectives. We therefore addressed the need for evidence-based information on current GHG emission levels, technically feasible mitigation outcomes related to the pathway to net zero, and, importantly, co-benefits and trade-offs at the management scale for policy development. Modeling provides a means of examining such compatibility for different policy objectives and for giving policy teams confidence in supporting specific combinations of on-farm measures. This modeling undertaking has generated new and comprehensive evidence for the tackling of multiple environmental pressures, e.g., climate change, water quality deterioration, and loss of biodiversity, at the management scale. While the novel modeling work reported herein examined the technically feasible ceiling of mitigation of agricultural GHG emissions that might be possible across England, using a large list of on-farm measures, there remains a research need for work with multiple stakeholders to examine and elicit a consensus on the viability of shortlists of measures for different farm systems, since implementation of fewer measures is less daunting for farmers and less demanding on challenged financial bottom-lines.

## Supplementary Information

Below is the link to the electronic supplementary material.

Supplementary file1 (DOCX 450 KB)

## Data Availability

The datasets generated and analyzed are available from the corresponding author on reasonable request.
