# Supplementary material for: Global warming potential of farming systems across England: possible mitigation and co-benefits for water quality and biodiversity
Source: Agron Sustain Dev. 2025 Apr 2;45(2):22. doi: 10.1007/s13593-025-01015-4 (PMC11965256; doi:10.1007/s13593-025-01015-4)
Supplement: Supplementary file 1 — Supplementary file1 (DOCX 450 KB) [file 13593_2025_1015_MOESM1_ESM.docx]

**Global Warming Potential of different farming systems across England: magnitude of mitigation possible using best management and co-benefits for water quality and biodiversity**

*^a^Net Zero and Resilient Farming, Rothamsted Research, North Wyke, Okehampton, Devon, EX20 2SB, UK.*

Yusheng Zhang^†^, Adrian L. Collins

†Corresponding author email address: [yusheng.zhang@rothamsted.ac.uk](mailto:yusheng.zhang@rothamsted.ac.uk)


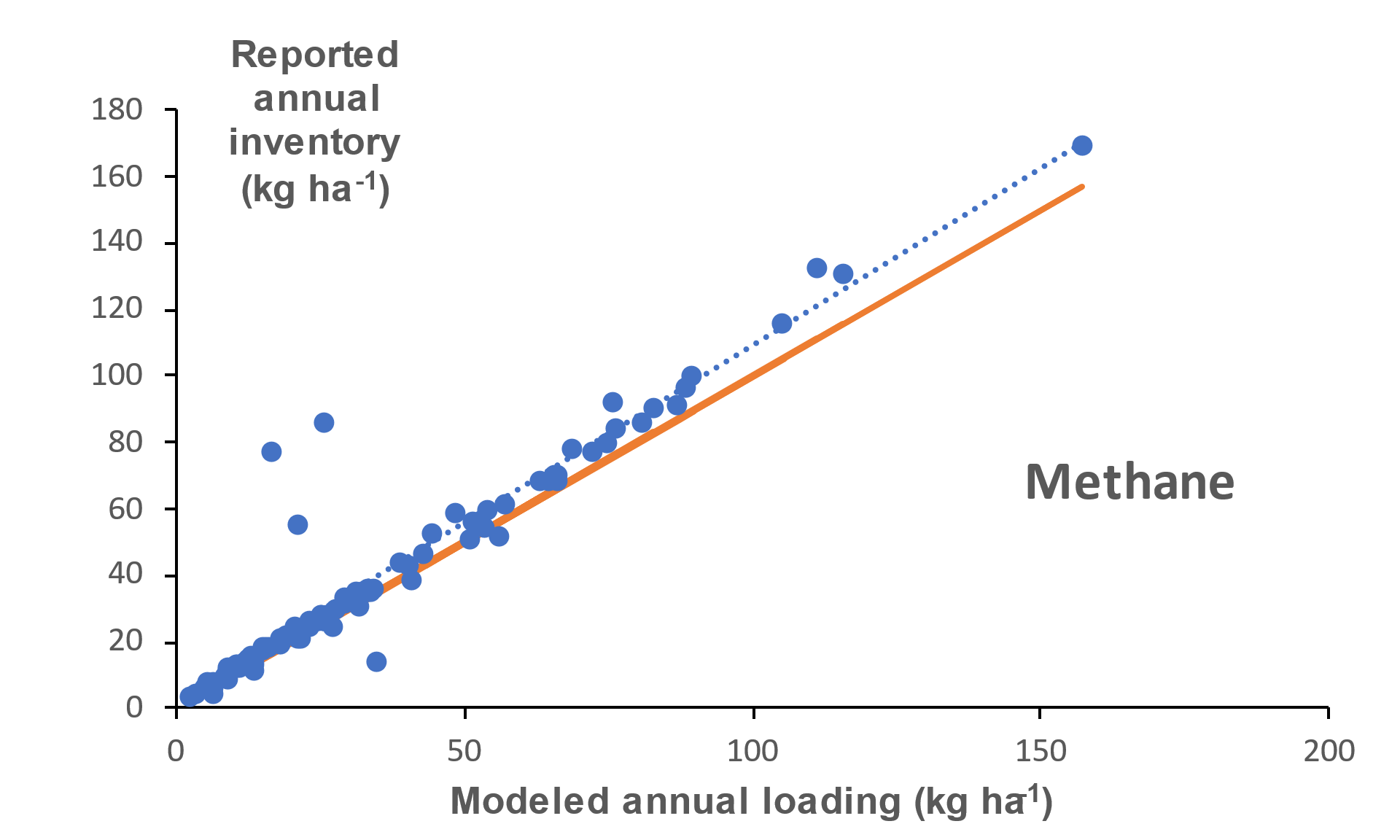
(a)


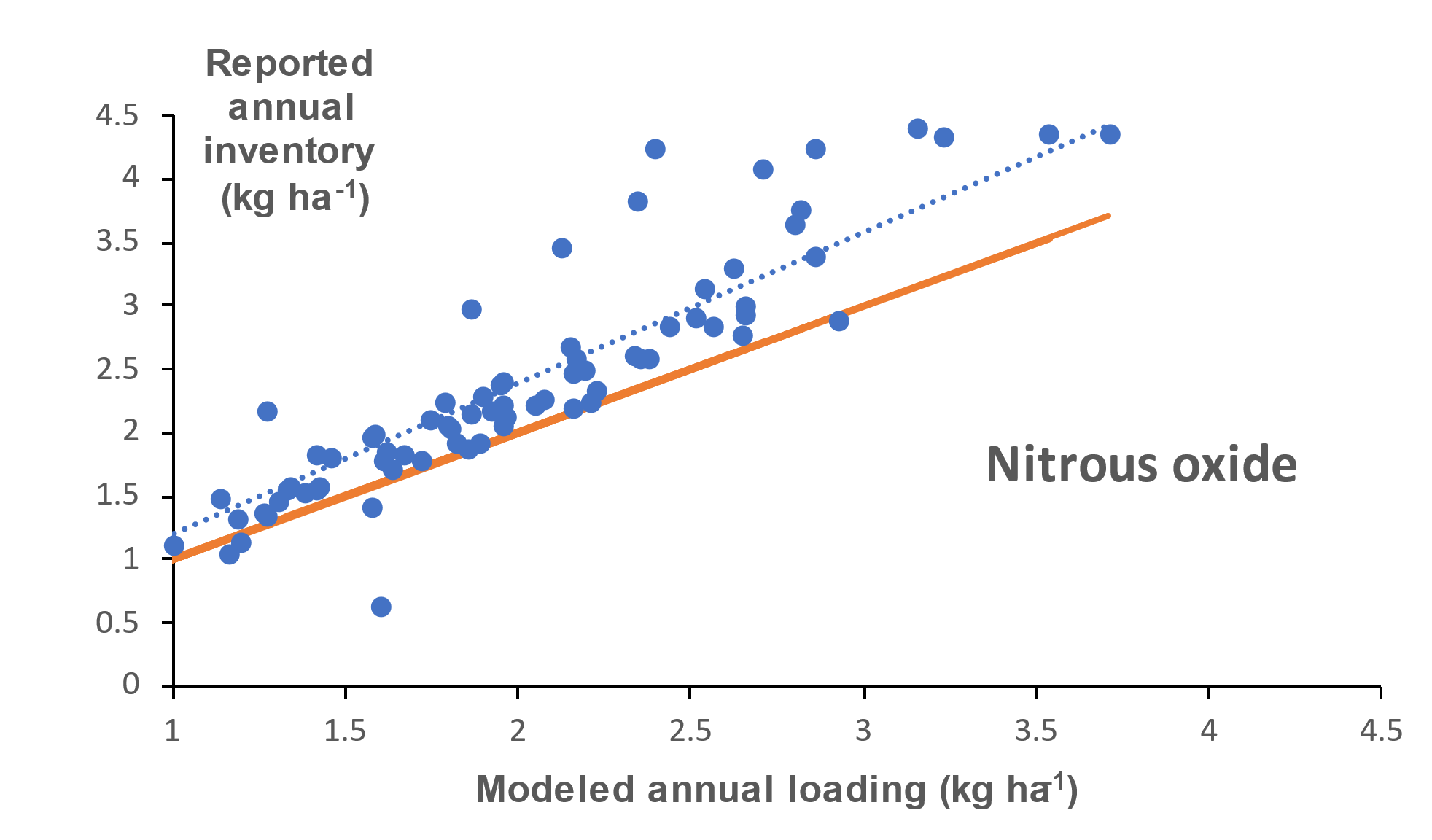
(b)

Fig. S1. Comparison of modeled GHG emissions against reported national inventories at WMC scale (straight line in red is the 1:1 line). Regression line equations are y = 1.0688x + 2.1786 with r^2^ = 0.91 for methane and y = 1.1896x + 0.0209 with r^2^ = 0.78 for nitrous oxide.


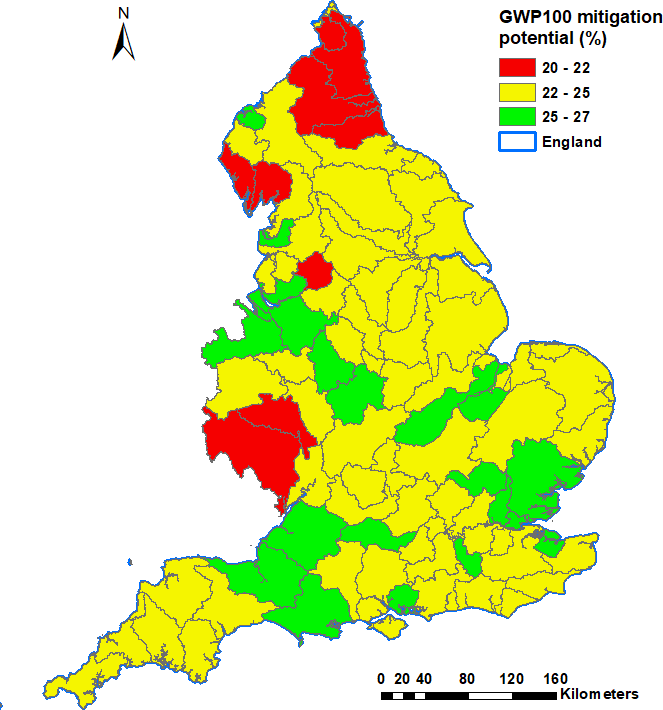


Fig. S2. Mapped maximum technical feasibility for the mitigation of GWP100 at WMC scale.

**
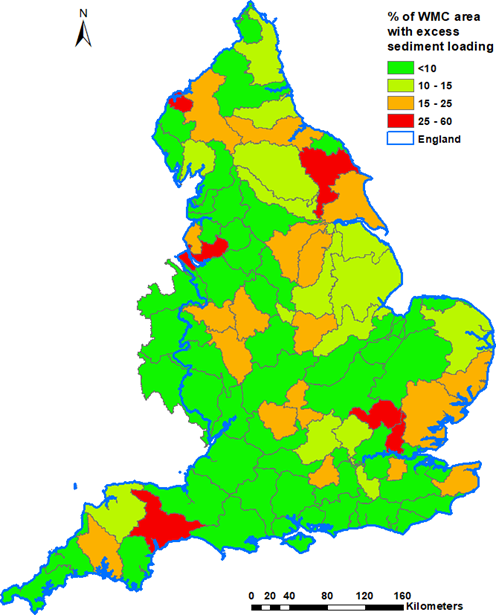
**

Fig. S3. Spatial distribution of excess sediment loadings at WMC scale.

**Table S1 Full list of mitigation measures included for the maximum technically feasible mitigation scenario modeling**

| Establish cover crops in the autumn |
| --- |
| Early harvesting and establishment of crops in the autumn |
| Cultivate land for crops in spring rather than autumn, retaining over-winter stubbles |
| Adopt reduced cultivation systems |
| Cultivate compacted tillage soils |
| Cultivate and drill across the slope |
| Leave autumn seedbeds rough |
| Manage over-winter tramlines |
| Establish in-field grass buffer strips |
| Establish riparian buffer strips |
| Loosen compacted soil layers in grassland fields |
| Allow grassland field drainage systems to deteriorate |
| Ditch management on arable land |
| Ditch management on grassland |
| Improved livestock through breeding |
| Use plants with improved nitrogen use efficiency |
| Fertiliser spreader calibration |
| Use a fertiliser recommendation system |
| Integrate fertiliser and manure nutrient supply |
| Do not apply manufactured fertiliser to high-risk areas |
| Avoid spreading manufactured fertiliser to fields at high-risk times |
| Use manufactured fertiliser placement technologies |
| Use nitrification inhibitors |
| Replace urea fertiliser to grassland with another form |
| Replace urea fertiliser to arable land with another form |
| Incorporate a urease inhibitor into urea fertilisers for grassland |
| Incorporate a urease inhibitor into urea fertilisers for arable land |
| Use clover in place of fertiliser nitrogen |
| Do not apply P fertilisers to high P index soils |
| Reduce dietary N and P intakes: Dairy |
| Reduce dietary N and P intakes: Pigs |
| Reduce dietary N and P intakes: Poultry |
| Adopt phase feeding of livestock: Dairy |
| Adopt phase feeding of livestock: Pigs |
| Reduce the length of the grazing day/grazing season |
| Extend the grazing season for cattle |
| Reduce field stocking rates when soils are wet |
| Move feeders at regular intervals |
| Construct troughs with concrete base |
| Increase scraping frequency in dairy cow cubicle housing |
| Additional targeted bedding for straw-bedded cattle housing |
| Washing down of dairy cow collecting yards |
| Frequent removal of slurry from beneath-slat storage in pig housing |
| Install air-scrubbers: mechanically ventilated pig housing |
| Install air-scrubbers: mechanically ventilated poultry housing |
| More frequent manure removal from laying hen housing with manure belt systems |
| In-house poultry manure drying |
| Increase the capacity of farm slurry stores to improve timing of slurry applications |
| Adopt batch storage of slurry |
| Install covers to slurry stores |
| Allow cattle slurry stores to develop a natural crust |
| Anaerobic digestion of livestock manures |
| Minimise the volume of dirty water produced (sent to dirty water store) |
| Minimise the volume of dirty water produced (sent to slurry store) |
| Compost solid manure |
| Site solid manure heaps away from watercourses/field drains |
| Store solid manure heaps on an impermeable base and collect effluent |
| Cover solid manure stores with sheeting |
| Use liquid/solid manure separation techniques |
| Use poultry litter additives |
| Manure Spreader Calibration |
| Do not apply manure to high-risk areas |
| Do not spread slurry or poultry manure at high-risk times |
| Use slurry band spreading application techniques |
| Use slurry injection application techniques |
| Do not spread FYM to fields at high-risk times |
| Incorporate manure into the soil |
| Fence off rivers and streams from livestock |
| Construct bridges for livestock crossing rivers/streams |
| Re-site gateways away from high-risk areas |
| Farm track management |
| Establish new hedges |
| Establish and maintain artificial wetlands - steading runoff |
| Irrigate crops to achieve maximum yield |
| Establish tree shelter belts around livestock housing |
| Calibration of sprayer |
| Fill/Mix/Clean sprayer in field |
| Avoid PPP application at high risk timings |
| Drift reduction methods |
| PPP substitution |
| Construct bunded impermeable PPP filling/mixing/cleaning area |
| Treatment of PPP washings through disposal, activated carbon or biobeds |
| Protection of in-field trees |
| Management of woodland edges |
| Management of in-field ponds |
| Management of arable field corners |
| Plant areas of farm with wild bird seed / nectar flower mixtures |
| Beetle banks |
| Uncropped cultivated margins |
| Skylark plots |
| Uncropped cultivated areas |
| Unfertilised cereal headlands |
| Unharvested cereal headlands |
| Undersown spring cereals |
| Management of grassland field corners |
| Leave residual levels of non-aggressive weeds in crops |
| Use correctly-inflated low ground pressure tyres on machinery |
| Locate out-wintered stock away from watercourses |
| Use dry-cleaning techniques to remove solid waste from yards prior to cleaning |
| Capture of dirty water in a dirty water store |
| Irrigation/water supply equipment is maintained and leaks repaired |
| Avoid irrigating at high risk times |
| Use efficient irrigation techniques (boom trickle, self closing nozzles) |
| Use high sugar grasses |
| Monitor and amend soil pH status for grassland |
| Increased use of maize silage |
| Improve livestock through genetic modification |
| Slurry acidification during storage |
| Slurry acidification at spreading |
| Install covers to slurry stores and burn off methane |
| Use feed additives to reduce enteric methane emissions |
